# Globular Tetramers of β_2_-Microglobulin Assemble into Elaborate Amyloid Fibrils

**DOI:** 10.1016/j.jmb.2009.03.066

**Published:** 2009-05-29

**Authors:** Helen E. White, Julie L. Hodgkinson, Thomas R. Jahn, Sara Cohen-Krausz, Walraj S. Gosal, Shirley Müller, Elena V. Orlova, Sheena E. Radford, Helen R. Saibil

**Affiliations:** 1 Department of Crystallography and Institute of Structural and Molecular Biology, Birkbeck College, Malet Street, London WC1E 7HX, UK; 2 Astbury Centre for Structural Molecular Biology, University of Leeds, Leeds LS2 9JT, UK; 3 Maurice E. Müller Institute, Biozentrum, Klingelbergstrasse, 50/70 CH-4056 Basel, Switzerland

**Keywords:** FTIR, Fourier transform infrared, 3D, three-dimensional, EM, electron microscopy, β_2_m, β_2_-microglobulin, STEM, scanning transmission electron microscopy, MPL, mass per unit length, protein misfolding, amyloid, cryo-EM, 3D reconstruction, STEM

## Abstract

Amyloid fibrils are ordered polymers in which constituent polypeptides adopt a non-native fold. Despite their importance in degenerative human diseases, the overall structure of amyloid fibrils remains unknown. High-resolution studies of model peptide assemblies have identified residues forming cross-β-strands and have revealed some details of local β-strand packing. However, little is known about the assembly contacts that define the fibril architecture. Here we present a set of three-dimensional structures of amyloid fibrils formed from full-length β_2_-microglobulin, a 99-residue protein involved in clinical amyloidosis. Our cryo-electron microscopy maps reveal a hierarchical fibril structure built from tetrameric units of globular density, with at least three different subunit interfaces in this homopolymeric assembly. These findings suggest a more complex superstructure for amyloid than hitherto suspected and prompt a re-evaluation of the defining features of the amyloid fold.

## Introduction

Despite the first identification of proteinaceous amyloid fibrils as the defining feature of clinical disease over 50 years ago,^1^ the full structure of amyloid fibrils remains unresolved. Although more than 25 protein and peptide sequences are involved in human amyloid diseases^2^ or in the formation of functional amyloid fibrils in prokaryotes and eukaryotes,^3^ it is now widely accepted that most polypeptide sequences can assemble into amyloid fibrils under suitable experimental conditions.^4,5^ The resulting fibrils appear to be built of a common core structure in which two or more long ribbons of β-sheet (protofilaments) assemble into flat or twisted fibrils.^6^ Accordingly, amyloid fibrils can be identified by generic properties such as the ability to bind dyes like Congo red and thioflavin T, with the former resulting in the characteristic green birefringence of amyloid fibres.^7^ In addition, all amyloid fibrils give rise to characteristic low-frequency absorption bands indicative of highly organised β-sheets visualised by Fourier transform infrared (FTIR)^8^ and a cross-β fibre diffraction pattern indicating that the constituent β-strands are oriented perpendicular to the fibril long axis.^9^ Consistent with a generic fold for amyloid, all such fibrils are recognised by the antibody WO1 and other ligands commonly found associated with amyloid fibrils in disease,^10,11^ irrespective of the length or sequence of the constituent polypeptide chains.

More recent studies of amyloid fibrils have focused on defining local structural information on β-sheet packing using X-ray fibre diffraction, solid-state NMR, and electron paramagnetic resonance spectroscopy.^12–15^ High resolution models for fibril core structures have recently been provided based on tightly packed β-sheets in three-dimensional (3D) crystals of short peptides,^16,17^ as well as assemblies built from parallel arrays of in-register β-strands in β-helical or serpentine arrangements.^6^ While these studies provide detailed information about local packing within the cross-β core, the larger scale assembly of amyloid fibrils is not well understood. For example, it is still unclear how protein subunits assemble into protofilaments and what determines protofilament assembly into fibrils. Because of the structural heterogeneity of fibril preparations seen by electron microscopy (EM) and atomic force microscopy, it is unlikely that these questions can be addressed by measurements on bulk samples. Different fibrils within a population contain different numbers of protofilaments and variable twists ranging from flat ribbons to tubular arrangements of protofilaments. This range of structures is typically seen within a single sample.^18–20^

In its native soluble form, the human protein β_2_-microglobulin (β_2_m) has a classical immunoglobulin fold and forms the non-covalently bound light chain of the class 1 major histocompatibility complex. During its normal catabolic cycle, β_2_m is degraded by the kidney. In patients with renal failure, the concentration of β_2_m in serum is increased by up to 60-fold, whereupon the full-length disulfide bonded protein self-associates into amyloid fibrils that accumulate in the musculo-skeletal system, causing dialysis-related amyloidosis.^21^ Like other soluble globular proteins involved in amyloidosis, native β_2_m is stable as a monomer in solution, and partial or full unfolding is required to initiate assembly into amyloid fibrils *in vitro*.^22^ While amyloid formation at neutral pH is a slow and inefficient process, seeding and/or addition of copper ions, detergents, or organic co-solvents can induce fibril formation at neutral pH.^23–25^ Under highly acidic conditions, the precursor monomeric protein is unfolded,^26^ and amyloid fibrils are rapidly formed in a nucleation-dependent mechanism.^27,28^ These fibrils display all the hallmarks of authentic amyloid, including nucleation-dependent polymerisation kinetics, Congo red birefringence, a cross-β fibre diffraction pattern, and the ability to bind serum amyloid P component, apolipoprotein E, glycosaminoglycans, and the generic anti-amyloid antibody WO1.^24,27,29^ They also show the same characteristic amide I band in FTIR spectra as fibrils formed *in vitro* at neutral pH, as well as those extracted from patient tissues, confirming their structural authenticity.^30^ FTIR analysis indicates a high β-sheet content in the fibrils, most likely involving a predominantly parallel arrangement of β-strands, in contrast with the anti-parallel arrangement of β-strands in the immunoglobulin fold of the native monomer.^31^

Here we describe the 3D structures of β_2_m amyloid fibrils obtained by cryo-EM and image processing by sorting fibril segments into homogeneous subsets. The 3D maps and mass analysis by scanning transmission electron microscopy (STEM) reveal a novel dimer-of-dimers repeat unit in a complex, hierarchical fibril assembly. We suggest that a globular repeat unit may be a common feature of amyloid fibrils formed from larger protein precursors. The results reveal a new view for amyloid, with a more elaborate superstructure than any previously described.

## Results

### Imaging and classification of amyloid fibril segments

**Fig. 1:**
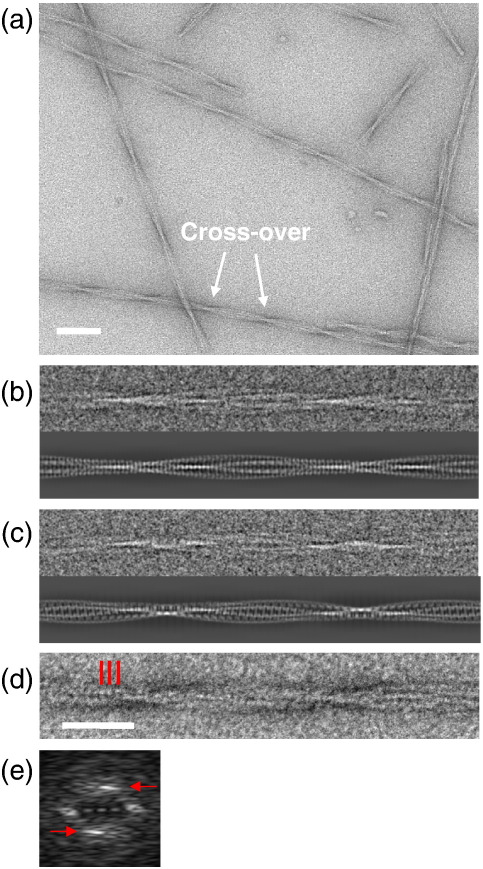
Helical and subunit repeats of β_2_m amyloid fibrils. (a) Negative-stain EM showing the helical twist in β_2_m fibrils. One cross-over repeat (180° turn) is labelled. (b) Cryo-EM class average (upper) and reprojection (lower) of the 3D reconstruction for a type A fibril. (c) Cryo-EM class average (upper) and reprojection (lower) for a type B fibril. (d) Negative-stain image of a cross-over with a marked region of subunit repeats. (e) Diffraction pattern from negatively stained fibrils with single sided staining. The marked layer line (arrows) indicates a spacing of 5.88 nm, corresponding to the subunit repeat. The layer line lies on an axis tilted by ∼ 7° from the fibre (vertical) axis, corresponding to the long-range helical twist of the fibrils. The intensity of the strong central diffraction was reduced by a factor of 10 so that it could be displayed together with the subunit layer lines. Scale bars represent 100 nm (a) and 50 nm (b–d).

**Fig. 2:**
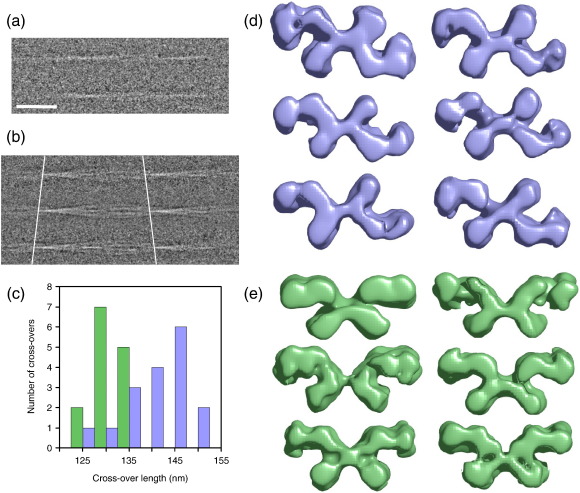
Variability in β_2_m fibril structure. (a) Class averages of C-type fibrils. (b) Three class averages of type A fibrils showing the large variation in cross-over spacing. (c) Histogram showing the frequency of cross-over repeats in the classes analysed (85% of the data). The A-type classes are shown in lilac, and the B-type classes are shown in green. (d and e) Cross sections of maps generated from different classes of (d) A-type and (e) B-type fibrils. Scale bar represents 50 nm (a and b). Map widths in (d) and (e) are ∼ 18–20 nm.

Images of β_2_m amyloid fibrils formed at pH 2.5, acquired by negative staining and cryo-EM, are shown in Fig. 1a. The images revealed long, twisted fibrils with a diameter of around 20 nm and a characteristic pattern of cross-over repeats. As found previously for other amyloid fibrils,^18,32^ β_2_m fibrils display a marked heterogeneity in repeat length (120–185 nm). To sort the fibrils into more uniform structural classes, each cross-over repeat unit was treated as a single particle.^33–35^ This approach revealed two main fibril morphologies, termed here as types A and B. Type A fibrils have polarity, as shown by the arrowhead features in the class average view in Fig. 1b, whereas type B fibrils are bipolar (Fig. 1c). Notably, hints of a globular subunit repeat can be seen in the class averages (Fig. 1b and c), which were determined by reference-free alignment and classification, with no repeat information imposed. This subunit repeat is clearly seen in raw negative stain images, whose power spectra show strong layer lines at spacings in the range of 5.2–6.5 nm, consistent with a subunit repeat of this size (Fig. 1d and e). A small population of fibrils with half the thickness of the type A and type B fibrils (10 nm) was also observed, termed type C (Fig. 2a). This finding, together with the observation that the thicker fibrils were occasionally split into two (data not shown), suggests that the fibrils are assembled from independent halves. Fibrils assembled at pH 7 showed morphologies similar to those generated at low pH, but were associated into bundles, precluding their further structural analysis (Supplementary Fig. 1).

### Three-dimensional reconstructions of amyloid fibrils

**Fig. 3:**
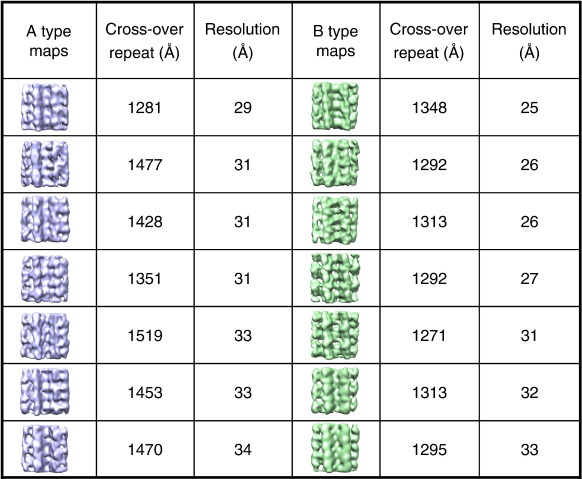
Selection of A-type (lilac) and B-type (green) maps showing side views, their cross-over repeat lengths, and resolutions at 0.5 Fourier shell correlation.

**Fig. 4:**
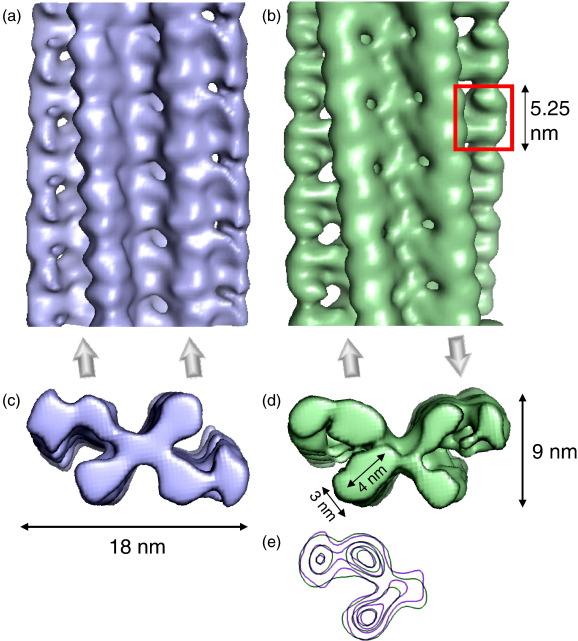
Three-dimensional reconstructions of the type A and type B forms of β_2_m fibrils. Side views of an A-type fibril (a) and a B-type fibril (b). The maps are contoured at a volume corresponding to an MPL of 53 kDa/nm. One dimeric density unit is indicated by a red box in (b). The directions of the half-fibrils are indicated by arrows below the maps. Cross sections of the A-type (c) and B-type fibrils (d) show that the structures are formed of crescent-shaped units stacked back-to-back. (e) Superposed contour plots of the A (lilac) and B (green) repeat units, showing that the two fibril types have the same underlying organisation that differs only in the orientation of the two stacks, either parallel or anti-parallel.

The intriguing substructure revealed by the images prompted us to undertake a 3D analysis of the fibril architecture. Of particular interest was the appearance of a subunit repeat, reminiscent of an earlier negative stain EM analysis of prion protein fibrils.^36^ However, this feature contrasts with current models for amyloid based on fibre diffraction, electron paramagnetic resonance, solid-state NMR, 3D crystals of small peptides, and cryo-EM images of fibrils formed from Aβ and other peptides.^6,14,37,38^ Using estimates of the long-range helical repeat and the subunit repeat determined from the images, 3D maps were calculated from the class average views. Reprojections of the maps compare well with the corresponding input class averages, showing the consistency of the maps with the input data (Fig. 1b and c). From a data set of 2000 repeats, 17 classes of the A-type structures and 14 classes of the B-type were selected, each with clear structural features, yielding a total of 31 individual 3D fibril maps (Fig. 2d and e). Side views, cross-over repeats, and resolution figures are tabulated for a selection of maps in Fig. 3. The resolution was estimated by comparing pairs of similar maps, using the 0.5 Fourier shell correlation criterion (Supplementary Fig. 2). Despite their structural variations, fibrils in the different classes have similar cross sections and bi-lobed globular repeats along the axial columns of density. The twist variation is most pronounced for the A-type classes where, for 85% of the data, the cross-over repeat distance fell into the range 128–152 nm (Fig. 2b and c). Outliers had cross-over repeats up to 185 nm, but their numbers were insufficient for classification and 3D reconstruction. For the B-type classes, the repeat is more constrained at 122–135 nm. A representative 3D map of each type is shown in more detail in Fig. 4, for which the resolutions are estimated as 29 Å (A-type) and 25 Å (B-type). The maps reveal a remarkably elaborate structure composed of two crescent-shaped units. A-type and B-type half-fibrils show a clear structural similarity when overlaid (Fig. 4e), consistent with the notion that the crescent-shaped half-fibrils are independent structural elements that can join back to back in either a parallel fashion (A-type; Fig. 4a and c) or an anti-parallel fashion (B-type; Fig. 4b and d). Each crescent contains three globular regions of density (about 3 nm × 4 nm in cross section), which represent constituent protofilaments (Fig. 4c and d). Most striking, however, is the complexity of the fibril architecture, with different interfaces between protofilaments both within and between the crescents. As shown in Fig. 2d and e, the A-type and B-type reconstructions also revealed considerable flexibility between the protofilaments and between the two crescents, adding to the heterogeneity of the samples obtained.

### STEM mass measurements

**Fig. 5:**
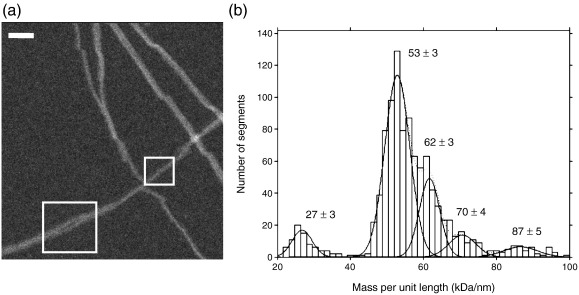
MPL measured by STEM. (a) STEM image showing fibrils with different masses per unit length. The boxes highlight 53-kDa/nm (lower left) and 27-kDa/nm (upper right) fibrils, respectively. Scale bar represents 57 nm. (b) Histogram showing the pooled data from 1030 STEM MPL measurements, with the component peaks fitted. Minor components in the samples with different morphologies may account for the smaller peaks at higher MPL.

To study the molecular packing of β_2_m into this homopolymer, STEM was used to determine mass per unit length (MPL) along the fibrils. The results revealed a major component at 53 ± 3 kDa/nm, as well as fibrils with half the width and a component at 27 ± 3 kDa/nm (Fig. 5). The former is consistent with the A-type and B-type fibrils, while the latter presumably represents the C-type fibrils. The component at 62 ± 3 kDa/nm may correspond to more tightly twisted fibrils, and minor components at higher mass may correspond to different fibril morphologies. The mass measurements of the major component indicate that there are 24 β_2_m monomers (monomer mass, 11.8 kDa) in each 5.25-nm repeat of the whole B-type fibril, or four monomers in each repeat of each protofilament. The alternating wide and narrow interfaces between density features along the protofilaments (Fig. 4a and b) suggest that the bi-lobed density corresponds to a head-to-head arrangement of each assembly unit. This tetrameric unit has 2-fold symmetry rather than 4-fold symmetry, so that the building block of the protofilament comprises four β_2_m molecules in a dimer-of-dimers arrangement.

**Fig. 6:**
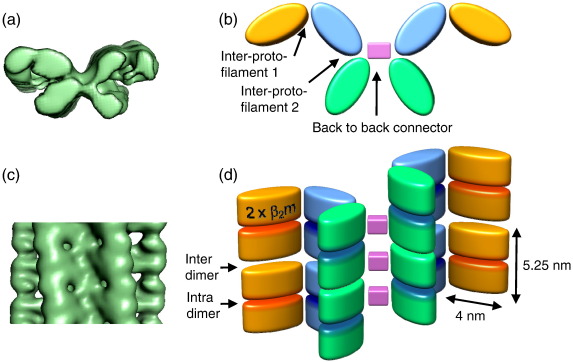
Schematic of subunit packing and interfaces in a B-type fibril. (a and b) Cross section and side view of a B-type fibril. (c and d) Schematic representation of the dimer-of-dimers subunit packing for the same fibril orientation; each elliptical cylinder corresponds to two β_2_m monomers. The outermost (orange) protofilaments in the model are disordered in the map, so that their full density is not reconstructed. It is possible, although unlikely, that pairs of protein subunits are threaded through all three protofilaments in one crescent with flexible hinge regions between the protofilaments.

The hierarchical fibril structure is explained schematically in Fig. 6. Three bi-lobed density units assemble into an extended crescent shape. Pairs of these crescent-shaped elements join back to back to form one layer of the fibril structure. Many copies of this assembly stack to form the slowly twisting helical fibril, in which columns of the globular building blocks constitute the six protofilaments of the fibril. In contrast to the generally accepted notion of a continuous cross-β core in amyloid fibrils,^4,6^ the globular substructure of these protofilaments gives them the appearance of a string of bi-lobed beads. The polarity of the half-fibrils arises from asymmetric connections between the protofilaments in the crescents.

## Discussion

Several models have been proposed for the structure of amyloid fibrils formed from intact β_2_m and its peptide fragments. A solid-state NMR study of a 22-residue peptide revealed pairs of β-strands packed in a parallel, staggered arrangement.^39^ A zipper-spine model has been proposed for fibrils formed from the intact protein, with residues 83–89 forming a central β-spine and with the rest of the sequence postulated to remain in a native-like state.^40^ Microcrystals of a short peptide (^82^NHVTLS^87^) show a simple cross-β ribbon, similar to the structures found in other peptide crystals of these presumed amyloid mimetics.^41^ Models based on crystal structures of β_2_m dimers and hexamers and others incorporating domain swapping or stacking of native subunits have also been proposed.^42–45^ None of these models is compatible with the 3D structure of the β_2_m amyloid presented here, a key feature of which is the globular dimer-of-dimers repeat. Consistent with the expectation of a non-native protein fold in the fibril, the density representing a dimer of β_2_m subunits (roughly 4 nm × 3 nm × 3 nm) cannot be accounted for by two copies of the native monomer (∼ 4 nm × 2 nm × 2 nm),^46^ stacked either axially or side by side, nor does a previously observed crystallographic dimer fit this density.^45^ This lack of fit is demonstrated by docking the atomic structures of the crystallographic dimer of the variant P32A,^45^ a domain-swapped dimer derived from molecular dynamics simulations,^44^ and a simple cross-β model or four native β_2_m monomers into the EM density of an extracted dimer-of-dimers unit (Supplementary Fig. 3). One way that the native-like monomers could fit is to be in staggered pairs (Supplementary Fig. 3e). However, in such an arrangement, interfaces completely different from those previously proposed or observed are required in order to fill the density, in addition to significant rearrangements of at least the surface loops to optimise packing and to avoid steric clashes. Moreover, such an arrangement does not yield the well-organised cross-β assembly expected in amyloid fibrils^9^ and observed for the fibrils analysed here^29^ and is incompatible with the proposed parallel arrangement of β-strands in these fibrils as indicated by FTIR.^31^ While the structure of β_2_m monomers within the fibril remains unresolved, refolding from the initially highly disordered monomeric state at pH 2.5^26^ upon amyloid assembly results in a globular, but non-native structure. This conformation is restrained by the native disulfide bond, which is required for fibrillation of monomeric β_2_m *in vitro* and *in vivo*.^22^

The most remarkable feature of the amyloid state of β_2_m is the dimer-of-dimers building block assembled into protofilaments that associate asymmetrically into the crescent-shaped units (Fig. 6). The resulting fibril architecture is discontinuous and very open, consistent with hydrogen-exchange protection factors of ∼ 10^3^–10^4^ along the sequence of β_2_m amyloid fibrils^47^ and the observation of pressure-induced fibril compaction.^48^ The elaborate fibril assembly reveals at least three non-equivalent subunit contacts. In the plane of each crescent, one protofilament makes the back-to-back connection to the equivalent protofilament in the opposite crescent, the central one links to a neighbour on either side, and the peripheral protofilament makes only one neighbour contact (Fig. 6b). Therefore, the β_2_m amyloid state does not simply arise from the same conformation in all subunits and, in marked contrast to the generic idea of amyloid as a continuous β-ribbon, the β_2_m protofilaments resemble a string of beads rather than a continuous β-sheet assembly. Importantly, the EM maps reveal that the globular repeat forms an integral part of the fibril structure and is not a folded “passenger” domain located externally to the fibril backbone, as seen, for example, in fibrils of Sup35 and Ure2p.^49,50^ This result is consistent with limited proteolysis experiments that revealed participation of ∼ 90 of the 99 residues in the fibril core and with hydrogen exchange measurements showing also an extensive protected core, with exchange kinetics suggestive of multiple environments for individual residues within the fibril structure.^47,51^ Such a complex architecture is also consistent with the observed importance of numerous stretches of the β_2_m polypeptide sequence in fibril formation.^41,52,53^ We note that a globular repeat is not unique to the β_2_m fibrils presented here, but has been suggested in earlier lower-resolution studies of fibrils formed from SH3 domains and the mammalian prion protein.^34,36^ Future models of amyloid will need to take into account the multiple intermolecular associations within a single homopolymer, most notably for fibrils formed from protein subunits, rather than peptide precursors.

## Methods

### Amyloid fibril preparation

Recombinant wild-type β_2_m was expressed in *Escherichia coli* and purified to homogeneity.^26^ The recombinant wild-type protein contained all 99 residues plus the N-terminal methionine, and the single disulfide bond was oxidised. Fibrils were formed by incubation of the protein (0.3–0.5 mg/ml) for 2–8 weeks at pH 2.5 in 25 mM sodium phosphate and 25 mM sodium acetate buffer containing 0.03% (wt/vol) sodium azide at 37 °C. At pH 7.0, fibrils were grown by elongation of heparin-stabilised seeds from fibrils formed at pH 2.5.^24^

### Transmission EM

β_2_m fibrils were used without dilution and negatively stained with 2% (wt/vol) uranyl acetate on thin carbon films supported on holey carbon coated EM grids. Microscopy was carried out on a Tecnai 10 (FEI, Eindhoven, NL) operating at 100 kV with a low electron dose and defocus values of 700–900 nm. For cryo-EM, the fibrils were vitrified on holey carbon support films. Cryo-EM was performed using a Tecnai F20 with a field emission gun operating at 200 kV. Low electron dose images were recorded on Kodak SO163 film at 29,000×, with defocus in the range 1–2.6 μm.

### Single-particle image processing

Cryo-EM images were digitised on a Zeiss SCAI photoscanner (ZI Imaging, Swindon, UK) with a pixel size of 7 μm. Cross-over repeats were selected by manually marking the cross-over positions using Ximdisp^54,55^ and were boxed out in SPIDER.^56^ Images were averaged to 0.35 nm/pixel at the specimen level and extracted into 1080 × 1080-pixel boxes for data processing. Defocus was determined from carbon adjacent to holes using Ctffind2,^54,57^ and image phases were corrected for the effects of the contrast transfer function using IMAGIC.^58^ Images were bandpass-filtered with a low-frequency cutoff between 70 and 20 nm (adjusted according to defocus) and a high-frequency cutoff of 1.16 nm and normalised. Images were aligned in SPIDER,^56^ with subsequent multivariate statistical analysis and classification of images in IMAGIC. An initial separation into the three structural types prior to classification was based on an analysis of the eigen images. Classes were all set to the same polarity. An iterative procedure of alignment, classification, and polarity reversal was performed until no classes of opposite polarity were obtained. Averaged power spectra of negative stain segments were obtained by multivariate statistical analysis and classification. The subunit repeat is easier to recognise in stained fibrils because of the higher contrast and as a consequence of single sided staining which avoids the overlap of upper and lower regions of the fibrils. This overlap obscures the subunit repeat pattern when the whole structure is seen in projection by cryo-EM because the repeats are out of register in the two halves of the fibrils.

### Three-dimensional reconstruction

Three-dimensional reconstructions using SPIDER were carried out on the best classes, those showing the clearest protofilament substructure, using helical symmetry.^56^ Two parameters had to be determined for the reconstruction: the cross-over repeat (Fig. 1a) and the subunit repeat (Fig. 1d). The cross-over repeat was determined by selecting a small region around one cross-over from the class average and by using cross correlation to determine the position of the other cross-over. The cross-over length was checked visually on the class average. Determination of the subunit repeat was more difficult, and several methods were used to verify the results. The starting point was the 5.88-nm repeat observed in diffraction patterns of negative stain images (Fig. 1e). Then, each map was reconstructed with a range of subunit repeats (4.5–7 nm), and the maps were examined to find the ones with the most distinct features, the highest contrast, and the best-matching reprojections (Supplementary Fig. 4). Based on these comparisons, a subunit repeat of 5.78 nm was used for the A-type maps, and a subunit repeat of 5.25 nm was used for the B-type maps. Short segments were reconstructed, and these were helically averaged to generate a full repeat so that reprojections could be compared to the class averages.

### Scanning transmission electron microscopy

Stock β_2_m fibril solutions were either diluted 20× to 100× in buffer or water and used immediately, or diluted 80× in an aqueous glutaraldehyde solution (final aldehyde concentration, 0.1%) and used after 5 min of incubation at room temperature. For mass measurement, 7 μl aliquots were adsorbed for 1 min to glow-discharged thin carbon films supported on holey carbon films on 200-mesh gold-plated copper grids. The grids were blotted, washed in 7–12 drops of quartz double-distilled water, plunge-frozen in liquid nitrogen, and freeze-dried at − 80 °C and 5 × 10^−^ ^8^ Torr overnight in the microscope. Tobacco mosaic virus (kindly supplied by Dr. R. Diaz-Avalos, Institute of Molecular Biophysics, Florida State University) adsorbed to a separate grid, was air-dried, and served as mass standard. A Vacuum Generators STEM HB-5 interfaced to a modular computer system (Tietz Video and Image Processing Systems GmbH, D-8035 Gauting, Germany) was used to record 512 × 512-pixel dark-field images. An accelerating voltage of 80 kV, a nominal magnification of 200,000×, and doses of approximately 400 e/nm^2^ were used for the mass measurements. Repeated low-dose scans were also recorded from some grid regions to assess beam-induced mass loss. The IMPSYS^59^ and MASDET^60^ program packages were used for evaluation. Fibril segments were selected in square boxes and tracked. The total scattering within an integration box following their length was then calculated, and the scattering contribution of the supporting carbon film was subtracted. Division by the segment length gave the MPL. MPL values were corrected for beam-induced mass loss, scaled to the MPL of tobacco mosaic virus, binned, displayed in histograms, and fitted by Gaussian curves. The measured MPL was not dependent on either the dilution method or the glutaraldehyde treatment, allowing all data sets to be merged and displayed in a single histogram for the final analysis.
