# Supplementary material for: Globular Tetramers of β2-Microglobulin Assemble into Elaborate Amyloid Fibrils
Source: J Mol Biol. 2009 May 29;389(1):48–57. doi: 10.1016/j.jmb.2009.03.066 (PMC2726924; doi:10.1016/j.jmb.2009.03.066)
Supplement: Supplementary materials [file mmc1.pdf]

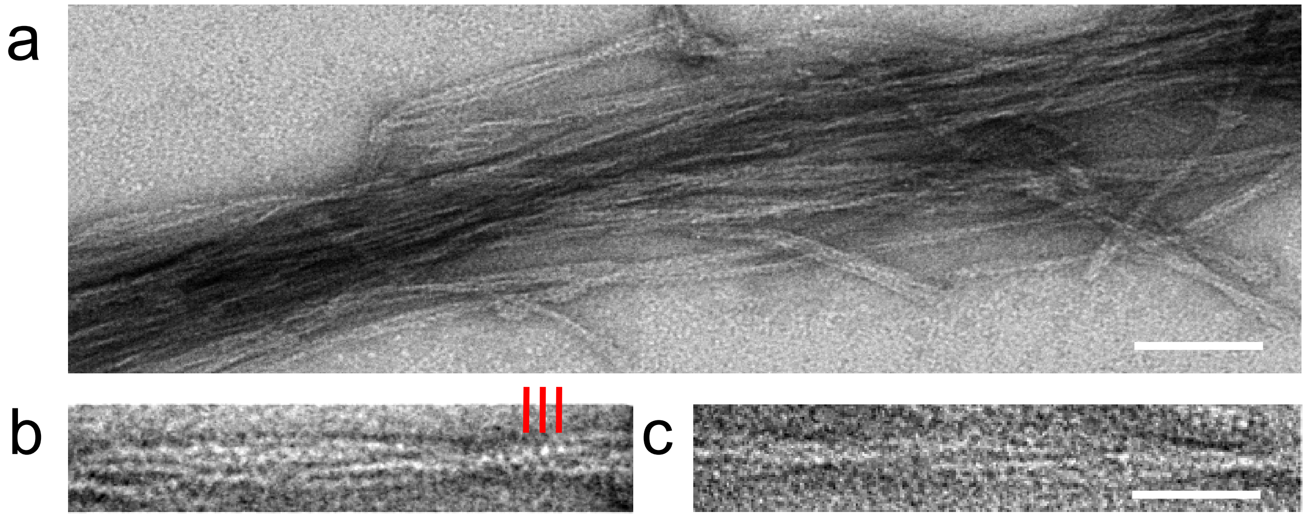

Supplementary figure 1 Comparison of wild-type β_2m fibrils formed at pH 7 and pH 2.5. (a) Twisted fibrils formed at pH 7 undergo extensive lateral bundling. Scale bar, 100 nm. (b) Enlargement of a pH 7 fibril, showing features of the subunit repeat along the protofilaments (red stripes). (c) Segment of a fibril formed at pH 2.5. Scale bar for b and c, 50 nm.

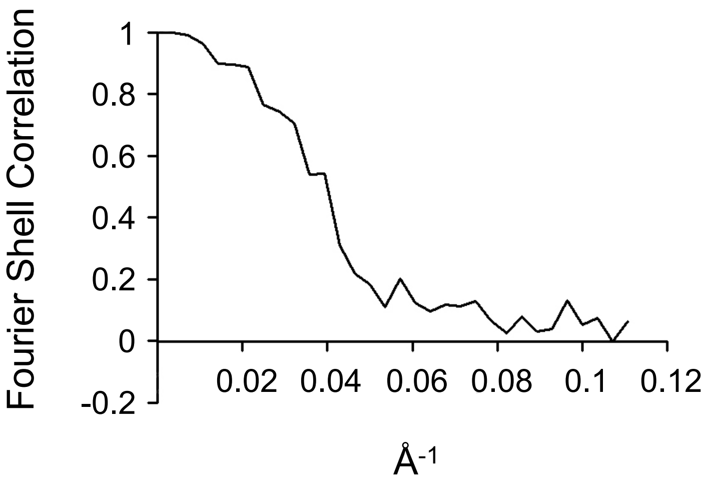

Supplementary figure 2. Fourier Shell Correlation between two B-type maps. The 0.5 correlation value corresponds to 25 Å.

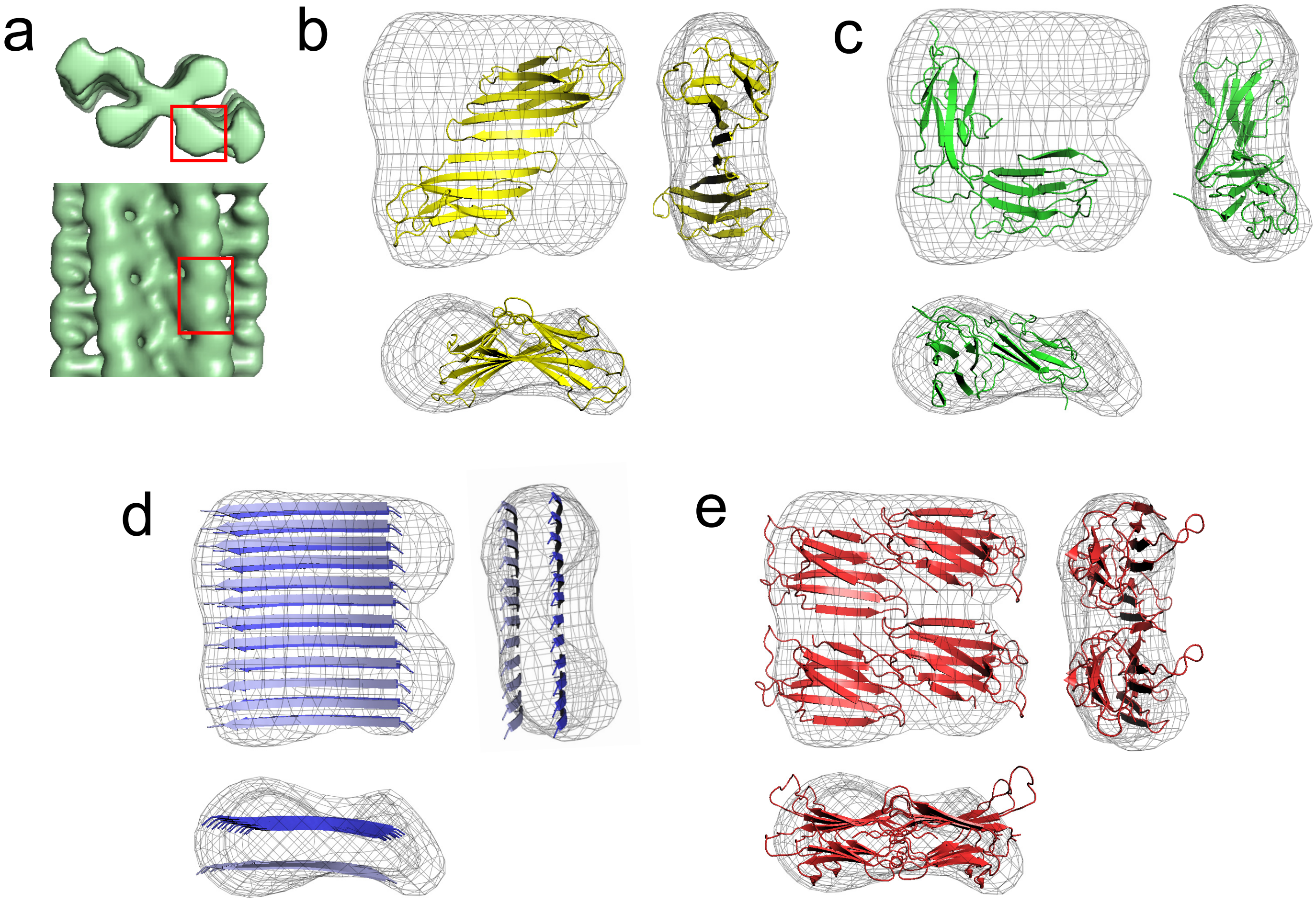

Supplementary Figure 3: Fitting of various models into the repeating structural unit of β_2m amyloid fibrils. **(a)** The subunit used for fitting is highlighted by a red box on the cryo-EM density map determined here. The repeating subunit of β_2m amyloid fibrils can not be described adequately by **(b)** the P32A β_2m dimer (PDB code 2F8O [40]) observed by X-ray crystallography, **(c)** a domain-swapped dimer derived from MD simulations [41] or **(d)** a generic cross-β amyloid model. A tetrameric structure assembled manually from native, wild-type β_2m monomers (PDB code 1LDS [43]) **(e)** leaves gaps between the stacked molecules and clashes in their side by side packing. Therefore, this model would require structural changes within the monomers to fit the density. In each case the structures have been fitted to produce cross-β characteristics as well as to match the density. The front, top and side view of one dimer-of-dimers repeat unit are shown for each model.

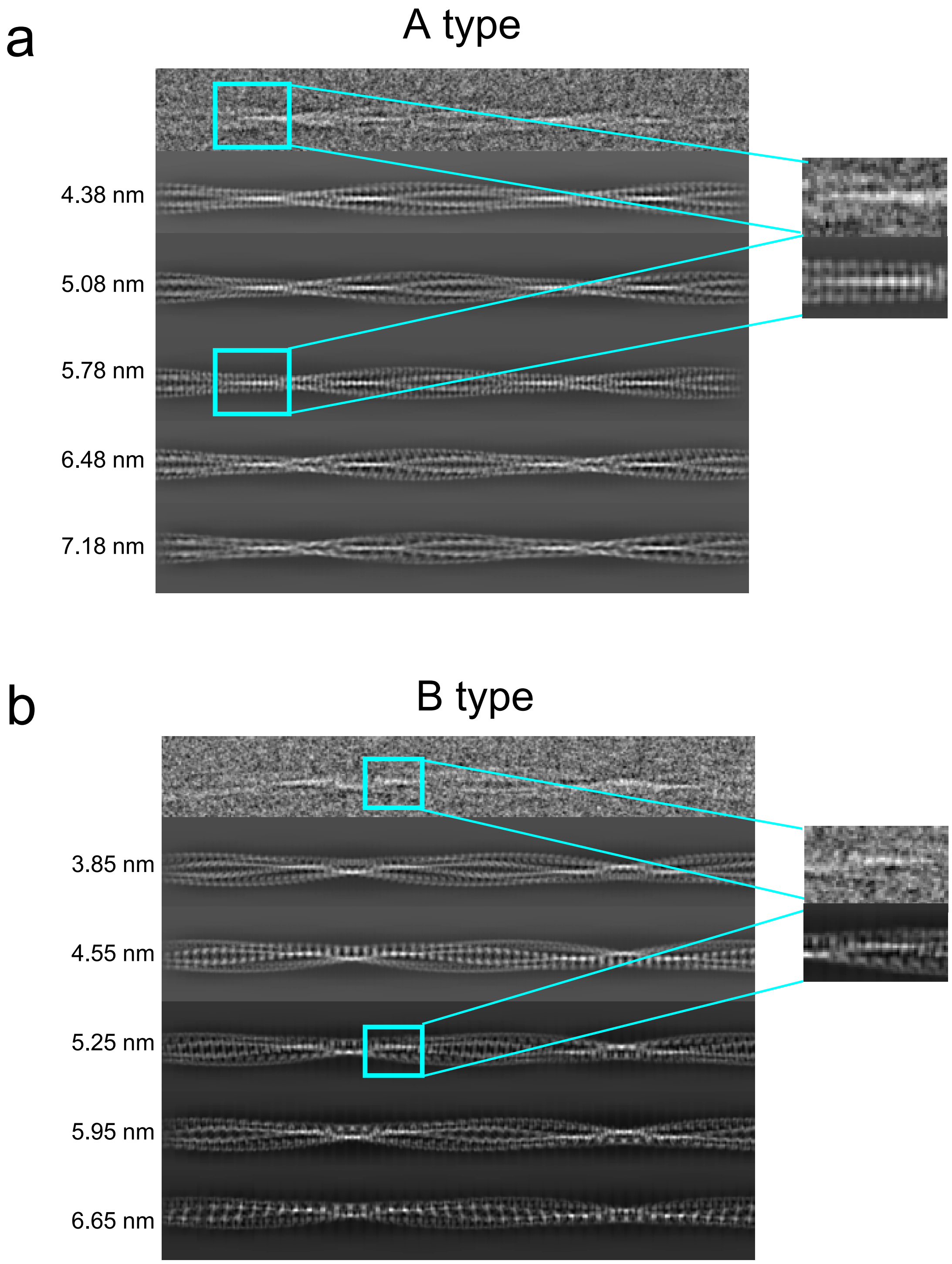

Supplementary figure 4 Analysis of subunit repeats. Maps were calculated with a range of different subunit repeats and their reprojections were compared to the input class average. (a) Class average and reprojections for an A-type fibril, with the subunit repeats shown on the left. The enlarged view shows the match of features in the class average and reprojection for a subunit repeat of 5.78 nm. (b) The same comparison for a B-type fibril, with the best match at 5.25 nm.
